# *SNPhood*: investigate, quantify and visualise the epigenomic neighbourhood of SNPs using NGS data

**DOI:** 10.1093/bioinformatics/btw127

**Published:** 2016-03-26

**Authors:** Christian Arnold, Pooja Bhat, Judith B. Zaugg

**Affiliations:** European Molecular Biology Laboratory (EMBL), Heidelberg, 69117, Germany

## Abstract

**Motivation:** The vast majority of the many thousands of disease-associated single nucleotide polymorphisms (SNPs) lie in the non-coding part of the genome. They are likely to affect regulatory elements, such as enhancers and promoters, rather than the function of a protein. To understand the molecular mechanisms underlying genetic diseases, it is therefore increasingly important to study the effect of a SNP on nearby molecular traits such as chromatin or transcription factor binding.

**Results:** We developed *SNPhood*, a user-friendly *Bioconductor* R package to investigate, quantify and visualise the local epigenetic neighbourhood of a set of SNPs in terms of chromatin marks or TF binding sites using data from NGS experiments.

**Availability and implementation:** SNPhood is publicly available and maintained as an R Bioconductor package at http://bioconductor.org/packages/SNPhood/.

**Contact:** judith.zaugg@embl.de

**Supplementary information:** Supplementary data are available at *Bioinformatics* online.

## 1 Introduction

To date, thousands of single nucleotide polymorphisms (SNPs) have been identified in genome-wide association studies (GWAS) to be associated with complex traits and diseases (www.genome.gov/gwastudies). The vast majority of these disease- or trait-associated SNPs lie in the non-coding part of the genome and are likely to affect regulatory elements (i.e. enhancers and promoters) rather than the function of a protein. Thus, to understand the molecular mechanisms underlying complex genetic traits and diseases, it is increasingly important to study the effect of SNPs on nearby molecular traits such as chromatin or transcription factor (TF) binding. Current workflows for analysing ChIP-Seq data typically involve peak calling, which summarises the signal of each binding event into two numbers: enrichment and peak size, and usually neglects additional factors like binding shape. However, when a set of regions of interest (ROI) is already at hand—e.g. GWAS SNPs, quantitative trait loci (QTLs), etc.—a comprehensive and unbiased analysis of the molecular neighbourhood of these regions, potentially in combination with genetic or allele-specific binding (ASB) analyses will be more suited to investigate the underlying (epi-)genomic regulatory mechanisms than simply comparing peak sizes. Currently, such analyses are often carried out ‘by hand’ using basic NGS tools and genome-browser like interfaces to visualise molecular phenotype data independently for each ROI. A tool for systematic analysis of the local molecular neighbourhood of ROI is currently lacking. To fill this gap, we developed *SNPhood*, an R/Bioconductor package to investigate, quantify and visualise the local epigenetic neighbourhood of ROI using chromatin or TF binding data from NGS experiments. It provides a set of tools that are largely complimentary to currently existing software for analysing ChIP-Seq data. Many functionalities of *SNPhood* have been used in our recent study to investigate the chromatin environment of H3K27ac QTLs, which has led to the conclusion that H3K27ac QTLs lie in the nucleosome free regions and show the same effect across multiple histone marks and TF binding (Grubert *et al.*, 2015). In addition, *SNPhood* supports ASB analyses, which is a powerful way for looking at the effect of genetic variants even within small sample sizes. We anticipate that it will be widely used for exploratory and quantitative functional genomics analysis of SNPs and other ROI using NGS data.

## 2 The *SNPhood* R package

### 2.1 Description and benchmark

*SNPhood* is an open-source R package (R Core Team, 2015) that is publicly available through Bioconductor (Huber *et al.*, 2015). It builds upon several of its established packages as well as ggplot2 (Wickham, 2009) for producing publication-quality visualisations.

**Fig. 1.**
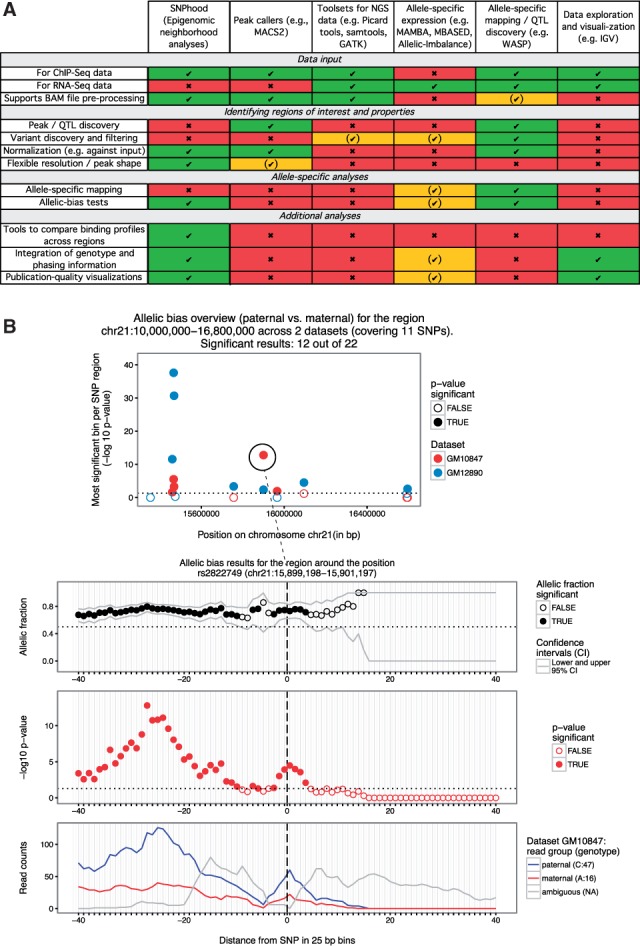
(**A**) *SNPhood overview.* Comparison and distinction of *SNPhood* with regard to commonly used tools for ChIP-Seq/RNA-Seq data. Green, yellow and red: Feature fully, partially or not supported, respectively. (**B**) Visualisation of an ASB region. All plots are a direct output of *SNPhood*. Upper panel: Overview of ASB for all SNPs within a particular genomic location on chr21 across two datasets based on an FDR threshold of 0.05. For each SNP, the most significant *p*-value (−log10 transformed) across 40 bins is shown. Lower panel: A detailed view around the SNP *rs2822749* (vertical line) for the individual GM10847. It summarises the allelic fraction estimate and confidence intervals (top), the -log10 *p*-value from the binomial tests within each bin (middle) and the read counts in each bin as well as the genotypes at the SNP position for the different alleles (bottom)

*SNPhood* comprises a set of easy-to-use functions to extract, normalise, quantify and visualise read counts or enrichment over input in the local neighbourhood of ROI (e.g. SNPs) across multiple samples (e.g. individuals). It's functionalities are largely complementary to and extend current tools used for ChIP-Seq data analysis (qualitative comparison shown in Fig. 1A). For instance, in contrast to peak callers that identify regions of enriched signal, *SNPhood* provides functionalities to perform in-depth analyses of the binding pattern on pre-defined ROI, group them according to their signal shape profiles and, if the data are provided, test for allele-specific and genotype-dependent binding patterns. The resolution of the binding pattern can be controlled by user-defined window and bin sizes, which define the local region surrounding the ROI and the size of individual bins within the neighbourhood for which read counts are quantified separately. Users can then choose from different analysis functions: (i) detection of allelic bias across ROI, for which we implemented a procedure that identifies the most significant bin within each region controlled by an empirically determined FDR, (ii) exploration and visualisation of genotype-dependent binding patterns including generation of publication-quality figures, or unsupervised clustering-based, and (iii), optionally, genotype-dependent comparisons and grouping of the binding pattern across ROI and samples. Methodological details and all functionalities can be found in the *SNPhood* Vignette (http://bioconductor.org/packages/release/bioc/vignettes/SNPhood/inst/doc/IntroductionToSNPhood.html).

As input, it requires (i) a set of BAM files (e.g. from ChIP-Seq), (ii) a list of genomic positions/ROI (e.g. GWAS-SNPs), and, optionally, (iii) corresponding genotype data. If available, *SNPhood* also allows background normalisation (e.g. input DNA) as employed by ChIP-Seq peak callers such as *MACS2* (Zhang *et al.*, 2008).

### 2.2 *SNPhood* application example

Here, we briefly illustrate some functionalities of *SNPhood* with a typical workflow example. For a comprehensive documentation of all functionalities, we refer the reader to the SNPhood workflow vignette: http://bioconductor.org/packages/release/bioc/vignettes/SNPhood/inst/doc/workflow.html. We start with a set of ROIs, in our case SNPs that have been identified as histone quantitative trait loci (hQTLs) within the Yoruba (YRI) population (Grubert *et al.*, 2015), and aim to determine how many YRI hQTLs also show ASB within the Caucasian population (CEU). To do so, we employed *SNPhood* for H3K27ac ChIP-Seq data from two CEU individuals (Kasowski *et al.*, 2013) around the hQTL SNPs to quantify their allelic bias. We found that 395 (33% of shared heterozygous SNPs) show significant ASB at an empirical FDR ∼5.4%. To investigate some of these ASB events in more detail, we used the function *plotAllelicBiasResultsOverview* for a high-level overview of the allelic bias across a chromosomal region. To visualise the binding pattern for specific ROI we employed the function *plotAllelicBiasResults* (Fig. 1B). This revealed that the selected region harbours two ASB peaks potentially affected by the same SNP. Further analyses would involve clustering of all ROI to identify common patterns, similar to what we performed in Grubert *et al.*, (2015).

Despite the name, *SNPhood* is flexible and can be applied to any ROI. We believe that it will be a helpful tool to generate new biological hypotheses by integrating molecular-phenotype data in an unbiased and position-specific manner.

## Supplementary Material

Supplementary Data
