# Supplementary material for: SNPhood: investigate, quantify and visualise the epigenomic neighbourhood of SNPs using NGS data
Source: Bioinformatics. 2016 Mar 26;32(15):2359–60. doi: 10.1093/bioinformatics/btw127 (PMC4965630; doi:10.1093/bioinformatics/btw127)
Supplement: Supplementary Data [file supp_32_15_2359__index.html]

SNPhood: investigate, quantify and visualise the epigenomic neighbourhood of SNPs using NGS data — SNPhood: investigate, quantify and visualise the epigenomic neighbourhood of SNPs using NGS data — SNPhood: investigate, quantify and visualise the epigenomic neighbourhood of SNPs using NGS data — Supplementary Data 

# *SNPhood*: investigate, quantify and visualise the epigenomic neighbourhood of SNPs using NGS data

## Supplementary Data

files

- Supplementary Data - pdf file
- Supplementary Data - pdf file
