# Supplementary material for: SNPhood: investigate, quantify and visualise the epigenomic neighbourhood of SNPs using NGS data
Source: Bioinformatics. 2016 Mar 26;32(15):2359–60. doi: 10.1093/bioinformatics/btw127 (PMC4965630; doi:10.1093/bioinformatics/btw127)
Supplement: Supplementary Data [file supp_btw127_SNPhood_comparison_supplement.pdf]

	SNPhood (Epigenomic neighbor-hood analyses)	Peak callers (e.g., MACS2[1])	Toolsets for NGS data analyses (e.g., Picard tools[2], samtools[3], GATK suite[4])	Allele-specific expression (e.g., MAMBA[5], Allelic-Imbalance[6] MBASED[7])	Allele-specific mapping / QTL discovery (e.g., WASP[8])	Data exploration and visuali-zation (e.g., Integrative Genomics Viewer – IGV[9])
	Data input					
Suitable for ChIP-Seq data	✔	✔	✔	✖	✔	✔
Suitable for RNA-Seq data	✖	✖	✔	✔	✔	✔
Supports (pre)processing of BAM files	✔	✔	✔	✖	(✔)	✖
	Identifying regions of interest and properties					
Peak / QTL discovery	✖	✔	✖	✖	✔	✖
Variant discovery and filtering	✖	✖	(✔)	(✔)	✔	✖
Normalization (e.g., against input)	✔	✔	✖	✖	✔	✖
Flexible resolution / peak shape	✔	(✔)	✖	✖	✖	✖
	Allele-specific analyses					
Allele-specific read mapping	✖	✖	✖	(✔)	✔	✖
Allelic-bias tests	✔	✖	✖	(✔)	✔	✖
	Additional analyses					
Tools to compare binding profiles across regions	✔	✖	✖	✖	✖	✖
Integration of genotype and phasing information	✔	✖	✖	(✔)	✖	✔
Publication-quality and user-adjustable visualizations	✔	✖	✖	(✔)	✖	✔

1) Zhang, Yong, et al. "Model-based analysis of ChIP-Seq (MACS)." Genome biology 9.9 (2008): R137.
2) http://broadinstitute.github.io/picard/
3) http://www.htslib.org/
4) https://www.broadinstitute.org/gatk
5) Pirinen, Matti, et al. "Assessing allele-specific expression across multiple tissues from RNA-seq read data." Bioinformatics (2015): btv074.
6) Gådin, Jesper R., et al. "AllelicImbalance: an R/bioconductor package for detecting, managing, and visualizing allele expression imbalance data from RNA sequencing." BMC bioinformatics 16.1 (2015): 194.
7) Mayba, Oleg, et al. "MBASED: allele-specific expression detection in cancer tissues and cell lines." Genome Biol 15.8 (2014): 405.
8) van de Geijn, Bryce, et al. "WASP: allele-specific software for robust molecular quantitative trait locus discovery." Nature Methods 12.11 (2015): 1061-1063.
9) Robinson, James T., et al. "Integrative genomics viewer." Nature biotechnology 29.1 (2011): 24-26. + Thorvaldsdóttir, Helga, James T. Robinson, and Jill P. Mesirov. "Integrative Genomics Viewer (IGV): high-performance genomics data visualization and exploration." Briefings in bioinformatics (2012): bbs017.
