# Supplementary material for: SNPhood: investigate, quantify and visualise the epigenomic neighbourhood of SNPs using NGS data
Source: Bioinformatics. 2016 Mar 26;32(15):2359–60. doi: 10.1093/bioinformatics/btw127 (PMC4965630; doi:10.1093/bioinformatics/btw127)
Supplement: Supplementary Data [file supp_btw127_SNPhood_Links.pdf]

SNPhood: Investigate, quantify and visualize the epigenomic neighborhood of SNPs using NGS data

1) Release version

Website (stable URL):
http://bioconductor.org/packages/SNPhood

Documentation: Introduction and Methodological Details:
https://www.bioconductor.org/packages/release/bioc/vignettes/SNPhood/inst/doc/IntroductionToSNPhood.html

Documentation: Workflow vignette:
https://www.bioconductor.org/packages/release/bioc/vignettes/SNPhood/inst/doc/workflow.html

2) Development version

Website (stable URL):
https://www.bioconductor.org/packages/devel/bioc/html/SNPhood.html

Documentation: Introduction and Methodological Details:
https://www.bioconductor.org/packages/devel/bioc/vignettes/SNPhood/inst/doc/IntroductionToSNPhood.html

Documentation: Workflow vignette:
https://www.bioconductor.org/packages/devel/bioc/vignettes/SNPhood/inst/doc/workflow.html

Please report any bug that you encounter as well as any feature request that you may have to SNPhood@gmail.com.
